# Epilepsy‐Related Psychosis in Drug‐Resistant Epilepsy With Nonconvulsive Status Epilepticus and Difficult Antipsychotic Titration: A Case Report

**DOI:** 10.1002/npr2.70153

**Published:** 2026-07-03

**Authors:** Wataru Omori, Ryo Hirabayashi, Kenichi Oga, Yoshikazu Masuda, Go Okada

**Affiliations:** ^1^ Department of Psychiatry NHO Kure Medical Center and Chugoku Cancer Center Kure Japan; ^2^ Department of Psychiatry Hiroshima University Hospital Hiroshima Japan; ^3^ Department of Psychiatry Senogawa Hospital Hiroshima Japan

**Keywords:** antipsychotic adverse effects, brexpiprazole, epilepsy, epilepsy‐related psychosis, forced normalization, nonconvulsive status epilepticus, tonic–clonic seizure

## Abstract

**Introduction:**

Psychosis in epilepsy may be difficult to diagnose when altered consciousness, antiseizure medication changes, and psychotropic adverse effects coexist.

**Case Presentation:**

We report a man in his late 50s with longstanding drug‐resistant epilepsy and recurrent psychosis. His documented convulsive seizures were classified as tonic–clonic seizures of unknown onset; epilepsy type and etiology could not be assigned. During admission for medication adjustment, reduced responsiveness and fluctuating alertness developed. EEG showed generalized rhythmic epileptiform activity at 2–3 Hz with evolution, and EEG/awareness improved after intravenous diazepam before fosphenytoin was introduced, supporting nonconvulsive status epilepticus. After treatment with fosphenytoin/phenytoin and improvement of nonconvulsive status epilepticus (NCSE), continuous EEG monitoring was performed to assess the phenytoin response and monitor electrographic recurrence. Serum antiseizure medication levels measured at multiple time points, together with MRI, blood tests, and the clinical course, made overt intoxication, acute structural or metabolic disease, and dementia with Lewy bodies less likely. After epileptiform activity attenuated and antipsychotics were withdrawn, psychosis re‐emerged without EEG evidence of ongoing status epilepticus. The course was interpreted as interictal psychosis with a possible alternative psychosis component. Aripiprazole improved psychosis but caused akathisia/dyskinesia, requiring cross‐titration to brexpiprazole. Levetiracetam reduction was limited by worsening EEG abnormalities.

**Discussion and Conclusion:**

This case emphasizes the need for a low threshold for EEG, structured diagnostic reasoning, and careful monitoring of pharmacokinetic interactions when treating psychosis in drug‐resistant epilepsy.

## Abstract 2

Electroclinical findings supported diazepam‐responsive nonconvulsive status epilepticus (NCSE) in an older patient with drug‐resistant epilepsy and psychosis. After phenytoin treatment, continuous EEG helped monitor recurrence. Later symptoms were most consistent with interictal psychosis with a possible alternative psychosis component, emphasizing structured diagnostic reasoning and careful pharmacologic monitoring.

## Graphical abstract

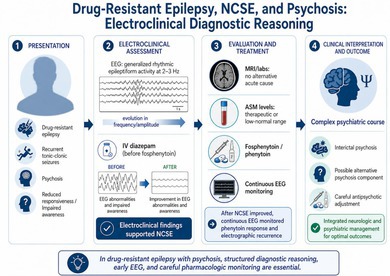

## 1 Introduction

Psychosis occurs in approximately 5%–6% of people with epilepsy [1]. It is usually classified as interictal, postictal, peri‐ictal, or associated with seizure suppression or electroencephalographic (EEG) normalization, often termed alternative psychosis or forced normalization [2].

Diagnostic precision requires neurological classification as well as psychiatric assessment. The International League Against Epilepsy (ILAE) frameworks encourage classification of seizure type, epilepsy type, and etiology when possible [3, 4], and the 2025 seizure classification emphasizes observable manifestations [5]. Nonconvulsive status epilepticus (NCSE) should be supported by standardized EEG description and, where possible, criteria such as the Salzburg criteria [6, 7].

We describe a patient with drug‐resistant epilepsy who developed NCSE, delirium‐like fluctuations, and medication‐related adverse effects during antipsychotic and antiseizure medication adjustment.

## 2 Case Presentation

The patient was a man in his late 50s with epileptic seizures since age 13 years. Despite treatment at several hospitals and vagus nerve stimulator implantation, he continued to have recurrent seizures under multiple antiseizure medications. Based on available records and caregiver descriptions, his documented convulsive seizures were best classified as tonic–clonic seizures of unknown onset; no ictal video‐EEG was available to determine focal versus generalized onset. Epilepsy type and etiology remained unclassified [3, 4, 5].

Approximately 2 years before the index admission, he became emotionally unstable and developed auditory hallucinations, running commentary, thought echo, persecutory delusions, and passivity phenomena. Agitation and disorganized behavior led to involuntary admission to a psychiatric emergency hospital, where paliperidone was introduced together with brexpiprazole. Psychotic symptoms improved sufficiently for discharge, but intermittent hallucinations and delusional thoughts persisted independently of overt seizures, supporting interictal psychosis. One year before the index admission, he was readmitted to the previous hospital because of generalized convulsive status epilepticus, which reportedly responded to intensive treatment. EEG records from that episode were unavailable, and we therefore could not determine whether convulsive status epilepticus had fully resolved or evolved into intermittent or persistent NCSE.

At first presentation to Hiroshima University Hospital, he showed mask‐like facies, stooped posture, bradykinesia, urinary incontinence, and excessive daytime sleepiness, in addition to residual hallucinations. These features were clinically attributed to parkinsonism and oversedation associated with paliperidone and brexpiprazole. Because outpatient adjustment was difficult, he was admitted to the psychiatric ward in early March of year X.

**FIGURE 1:**
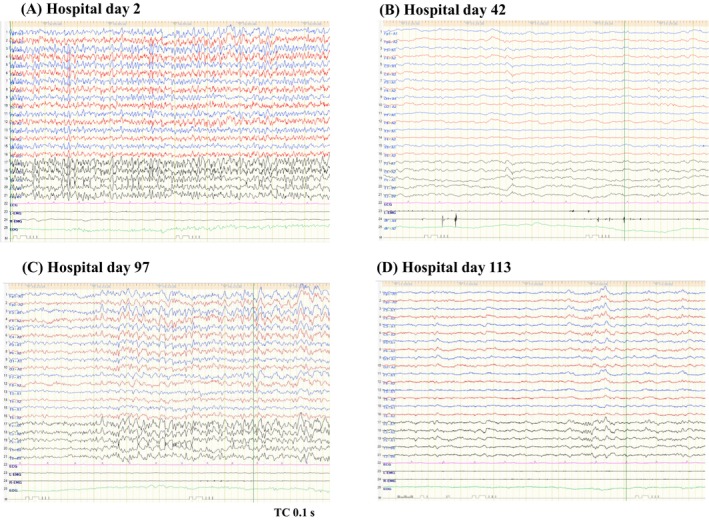
Representative EEG recordings during hospitalization. Scalp EEG electrodes were placed according to the international 10–20 system and displayed in an A1/A2 referential montage. (A) Hospital day 2: Generalized rhythmic epileptiform activity at 2–3 Hz was observed with reduced responsiveness and fluctuating alertness; EEG abnormalities and awareness improved after intravenous diazepam administered before fosphenytoin, supporting NCSE. (B) Hospital day 42: No prominent epileptiform discharges. (C) Hospital day 97: Epileptiform discharges re‐emerged after levetiracetam dose reduction. (D) Hospital day 113: Epileptiform abnormalities improved after increasing levetiracetam and adding lamotrigine. The time constant displayed in the figure is 0.1 s. EEG, electroencephalogram; NCSE, nonconvulsive status epilepticus.

On admission, he was alert but slowed. Paliperidone 6 mg/day, brexpiprazole 2 mg/day, clobazam, sodium valproate, levetiracetam, and lacosamide were continued. Soon afterward, he developed reduced responsiveness and fluctuating alertness. Medication‐related sedation and delirium were considered, but EEG on hospital day 2 demonstrated generalized rhythmic epileptiform activity at 2–3 Hz that persisted throughout the recording, evolved in frequency and amplitude, and was not clearly attenuated by stimulation (Figure 1A). Before fosphenytoin was introduced, intravenous diazepam was administered; both EEG abnormalities and impaired awareness improved. These electroclinical findings supported NCSE and were considered consistent with the Salzburg criteria [6, 7]. Continuous video‐EEG and density spectral array were unavailable; therefore, the diagnosis was not presented as beyond all uncertainty.

Subsequently, intravenous fosphenytoin was administered over 2 days, followed by oral phenytoin titrated to 300 mg/day. Awareness improved as epileptiform activity attenuated. After the acute NCSE episode settled, continuous EEG monitoring was performed to assess the effect of phenytoin and to monitor for electrographic recurrence. Paliperidone and brexpiprazole were discontinued. Sodium valproate was briefly increased but stopped because of hyperammonemia. During recovery, fluctuating lethargy, agitation, and disorientation were interpreted as delirium during recovery from NCSE, possibly compounded by polypharmacy.

Serum antiseizure medication levels, measured at multiple time points, did not suggest overt intoxication: levetiracetam 35.2 μg/mL at 3000 mg/day, phenytoin 13.4 μg/mL at 200 mg/day and 17.6 μg/mL at 300 mg/day (therapeutic range, 10–20 μg/mL) [8], valproic acid 75.0 μg/mL at 600 mg/day, lacosamide 3.5 μg/mL at 400 mg/day, and lamotrigine 3.0 μg/mL at 25 mg/day before breakfast.

Additional anonymized hospital‐day‐based clinical‐course data, including serial EEG findings and serum antiseizure medication levels, are provided in Table S1.

Brain MRI showed no definite structural abnormalities, including significant atrophy or ischemic changes. Blood tests showed no inflammatory, metabolic, or autoimmune findings explaining the altered mental status. After NCSE improved, the Mini‐Mental State Examination score was 28/30. There was no REM sleep behavior disorder, visual hallucinations, persistent parkinsonism, or autonomic dysfunction. Dopamine transporter SPECT was not performed; dementia with Lewy bodies was therefore less likely but not formally excluded.

Approximately 7 weeks after admission, while seizures were reasonably controlled and EEG no longer showed continuous epileptiform activity (Figure 1B), psychosis recurred with derogatory voices, thought echo, thought insertion, and commanding voices. There were no clinical seizures, and EEG did not show ongoing NCSE. Because symptoms re‐emerged after epileptiform activity attenuated and antipsychotics were withdrawn, the episode was interpreted as interictal psychosis [9, 10] with a possible alternative psychosis component, not definite forced normalization [11, 12]. Aripiprazole 12 mg/day was started and titrated to 18 mg/day, with improvement.

At higher aripiprazole doses, prominent akathisia and orofacial dyskinesia developed. Clonazepam partially alleviated dyskinesia but appeared to cause disinhibited behavior and was stopped. Propranolol improved akathisia. Aripiprazole was gradually reduced, and brexpiprazole was reintroduced at 1 mg/day and titrated to 2 mg/day during cross‐titration. As aripiprazole was discontinued, dyskinesia and akathisia improved, and propranolol could be stopped.

Because levetiracetam‐related psychiatric adverse effects were suspected [13], levetiracetam was reduced from 3000 to 2500 mg/day, but epileptiform EEG abnormalities increased without clear seizures (Figure 1C), limiting further reduction. Levetiracetam was increased to 2750 mg/day, and lamotrigine was introduced and titrated to 25 mg/day; epileptiform abnormalities improved (Figure 1D). At discharge on hospital day 142, his regimen was lacosamide 400 mg/day, levetiracetam 2750 mg/day, phenytoin 300 mg/day, lamotrigine 25 mg/day, and brexpiprazole 2 mg/day. He was oriented, had only occasional attenuated voices, and showed minimal extrapyramidal symptoms.

**FIGURE 2:**
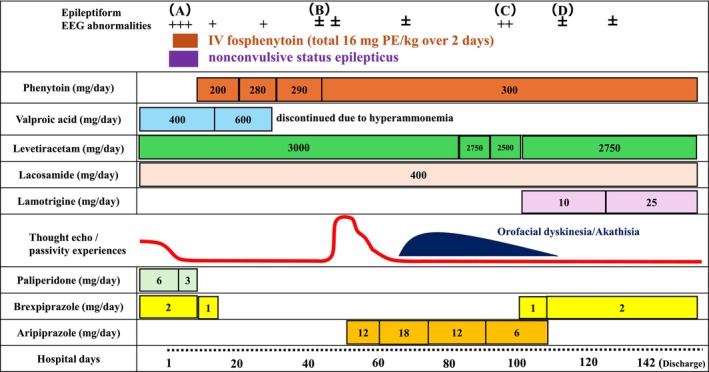
Clinical course during hospitalization. The top row shows semiquantitative grading of epileptiform EEG abnormalities (−, absent; ±, minimal; + to +++, increasing severity), together with the timing of intravenous fosphenytoin administration and the clinically suspected period of NCSE. Intravenous fosphenytoin (total 16 mg PE/kg over 2 days) was administered, followed by oral phenytoin titration. Daily doses of antiseizure medications and antipsychotics are shown in mg/day. Valproic acid was discontinued because of hyperammonemia. The red trace indicates the severity of psychotic symptoms (thought echo and passivity experiences), and the blue shaded area indicates the period of orofacial dyskinesia/akathisia. Letters (A–D) mark the time points at which representative EEG recordings were obtained. EEG, electroencephalogram; NCSE, nonconvulsive status epilepticus; PE, phenytoin equivalents.

The longitudinal clinical course, including medication changes, EEG findings, psychiatric symptoms, and adverse events, is summarized in Figure 2. The diagnostic interpretation across the major clinical phases is summarized in Table 1.

**TABLE 1:** Structured diagnostic interpretation across the clinical course.

Clinical phase	Working interpretation	Key supporting findings	Competing explanations or limitations
Before index admission	Interictal psychosis	Hallucinations, thought echo, delusions, and passivity phenomena persisted independently of overt seizures	Drug‐induced psychosis and levetiracetam‐related vulnerability remained possible; MRI, laboratory findings, MMSE, and clinical features argued against acute organic disease or dementia with Lewy bodies as the primary explanation
Admission and acute treatment	Electroclinically supported NCSE, with medication‐related sedation/delirium as competing explanations	Fluctuating alertness and psychomotor slowing; 2–3 Hz rhythmic epileptiform activity with evolution; and improvement in EEG abnormalities and awareness after intravenous diazepam before fosphenytoin	Continuous video‐EEG with density spectral array was unavailable during the acute diagnostic period; therefore, NCSE was supported electroclinically but not presented as certain beyond the available data
Post‐NCSE monitoring after phenytoin introduction	Assessment of phenytoin response and surveillance for electrographic recurrence	After the acute NCSE episode settled, continuous EEG monitoring was performed to assess the effect of phenytoin and to monitor for electrographic recurrence	This monitoring supported treatment assessment and recurrence surveillance; it was not used as the sole basis for the initial NCSE diagnosis
Recovery phase	Delirium during recovery from NCSE and polypharmacy	Alternating lethargy, agitation, and disorientation after attenuation of continuous epileptiform activity	Overt phenytoin toxicity was less likely based on therapeutic levels, but antipsychotic/benzodiazepine effects and polypharmacy remained possible
Psychosis recurrence after EEG improvement	Interictal psychosis with a possible alternative psychosis component	Thought echo, passivity phenomena, and hallucinations recurred without clinical seizures or EEG evidence of ongoing NCSE	Definite forced normalization was not claimed; recurrent interictal psychosis remained the core diagnosis
Late titration phase	Medication adverse effects and seizure vulnerability	Aripiprazole‐related akathisia/dyskinesia; increased epileptiform abnormalities after levetiracetam reduction	Phenytoin enzyme induction may have altered antipsychotic and antiseizure medication exposure despite therapeutic measured levels

## 3 Discussion and Conclusion

This case illustrates how NCSE, delirium, medication adverse effects, and psychosis of epilepsy can overlap. The early episode was supported as NCSE by generalized rhythmic epileptiform activity at 2–3 Hz with evolution, lack of clear attenuation by stimulation, and improvement of EEG abnormalities and awareness after intravenous diazepam, which was administered before fosphenytoin. After the acute episode settled, continuous EEG monitoring was also performed to assess phenytoin response and monitor for electrographic recurrence. Because continuous video‐EEG with density spectral array was unavailable during the acute diagnostic period, we avoided presenting the initial diagnosis as beyond all uncertainty.

Differential diagnosis was central. Drug‐induced sedation and delirium were plausible because of parkinsonism, sleepiness, benzodiazepine exposure, hyperammonemia, and polypharmacy. However, the EEG pattern and response to diazepam supported NCSE rather than medication effects alone. Measured phenytoin levels made overt toxicity less likely. MRI and blood tests did not reveal acute structural, inflammatory, metabolic, or autoimmune abnormalities. Dementia with Lewy bodies was considered, but hallucinations were predominantly auditory, parkinsonism improved after antipsychotic adjustment, cognition was relatively preserved, and REM sleep behavior disorder, visual hallucinations, and autonomic dysfunction were absent [14]. Without dopamine transporter SPECT, DLB could not be formally excluded.

The recurrent psychosis was best regarded as interictal psychosis with a possible alternative psychosis component. Symptoms had appeared before admission and persisted outside clear seizure clusters, fitting interictal psychosis. During admission, psychosis recurred after attenuation of continuous epileptiform activity and withdrawal of antipsychotics, without EEG evidence of ongoing NCSE. This temporal pattern is suggestive of alternative psychosis but does not prove forced normalization.

Pharmacological interpretation was also complicated. Aripiprazole improved psychosis but caused akathisia and orofacial dyskinesia. Brexpiprazole was better tolerated after cross‐titration, but this single case cannot establish comparative efficacy. Levetiracetam reduction was attractive because of suspected psychiatric adverse effects, yet EEG worsening limited dose reduction. Levetiracetam‐related psychosis is more likely in patients with status epilepticus, previous psychosis, and concomitant phenytoin, whereas lamotrigine co‐therapy may be protective [15].

Phenytoin may have influenced the course beyond seizure control. Measured levels were therapeutic, making overt toxicity less likely, but concentration‐dependent adverse effects may still be context‐dependent. As an enzyme inducer, phenytoin can reduce concentrations of several psychotropic and antiseizure medications, including CYP3A substrates [16], complicating interpretation of both seizure control and psychiatric symptoms. Neuropsychiatric manifestations of phenytoin intoxicationhave also been reported [17, 18].

The main limitations are the single‐case design, incomplete EEG quantification, lack of continuous video‐EEG with density spectral array during the acute diagnostic period, therapeutic drug monitoring at multiple but non‐systematic time points, absence of standardized psychiatric or extrapyramidal symptom scales, and lack of dopamine transporter SPECT or formal neuropsychological evaluation. Even so, the case provides practical lessons: obtain EEG early when mental status changes cannot be explained by medication effects alone; distinguish interictal psychosis from possible alternative psychosis cautiously; and plan antipsychotic titration with attention to seizure risk [19, 20] and pharmacokinetic interactions [16].

## Author Contributions

W.O. drafted and revised the manuscript; R.H. managed the patient and collected data; K.O., Y.M., and G.O. contributed to patient management and manuscript revision; all authors approved the final version of the manuscript.

## Funding

The authors have nothing to report.

## Ethics Statement

The patient received clinical care at the Department of Psychiatry, Hiroshima University Hospital. This case report was reviewed and approved by the institutional review board of the NHO Kure Medical Center (approval no. 2025‐91).

## Consent

Written informed consent for publication was obtained from the patient.

## Conflicts of Interest

W.O. has received lecture fees and chairperson honoraria from Daiichi Sankyo Co. Ltd., Eisai Co. Ltd., Sumitomo Pharma Co. Ltd., and Otsuka Pharmaceutical Co. Ltd. W.O. has also received contract research funding for clinical trials from Otsuka Pharmaceutical Co. Ltd. The other authors declare no conflicts of interest.

## Supporting information


**Table S1:** EEG recordings: de‐identified EEG monitoring dataset.

## Data Availability

The anonymized clinical‐course data underlying this case report are provided in Table S1. Additional data that support the findings of this study are available from the corresponding author upon reasonable request and subject to institutional and ethical restrictions. Full raw clinical records, source EEG files, and other potentially identifiable materials are not publicly available because this is a single‐patient case report and disclosure could compromise patient privacy.

## References

[1] M. J. Clancy , M. C. Clarke , D. J. Connor , M. Cannon , and D. R. Cotter , “The Prevalence of Psychosis in Epilepsy: A Systematic Review and Meta‐Analysis,” BMC Psychiatry 14 (2014): 75. 24625201 10.1186/1471-244X-14-75 PMC3995617

[2] A. M. Kanner and A. M. Rivas‐Grajales , “Psychosis of Epilepsy: A Multifaceted Neuropsychiatric Disorder,” CNS Spectrums 21, no. 3 (2016): 247–257. 27322691 10.1017/S1092852916000250

[3] R. S. Fisher , J. H. Cross , J. A. French , et al., “Operational Classification of Seizure Types by the International League Against Epilepsy: Position Paper of the ILAE Commission for Classification and Terminology,” Epilepsia 58, no. 4 (2017): 522–530. 28276060 10.1111/epi.13670

[4] I. E. Scheffer , S. Berkovic , G. Capovilla , et al., “ILAE Classification of the Epilepsies: Position Paper of the ILAE Commission for Classification and Terminology,” Epilepsia 58, no. 4 (2017): 512–521. 28276062 10.1111/epi.13709 PMC5386840

[5] S. Beniczky , E. Trinka , E. Wirrell , et al., “Updated Classification of Epileptic Seizures: Position Paper of the International League Against Epilepsy,” Epilepsia 66, no. 6 (2025): 1804–1823. 40264351 10.1111/epi.18338 PMC12169392

[6] M. Leitinger , E. Trinka , E. Gardella , et al., “Diagnostic Accuracy of the Salzburg EEG Criteria for Non‐Convulsive Status Epilepticus: A Retrospective Study,” Lancet Neurology 15, no. 10 (2016): 1054–1062. 27571157 10.1016/S1474-4422(16)30137-5

[7] L. J. Hirsch , M. W. K. Fong , M. Leitinger , et al., “American Clinical Neurophysiology Society's Standardized Critical Care EEG Terminology: 2021 Version,” Journal of Clinical Neurophysiology 38, no. 1 (2021): 1–29. 33475321 10.1097/WNP.0000000000000806 PMC8135051

[8] Japanese Society of Neurology , Clinical Practice Guidelines for Epilepsy 2018 (Igaku‐Shoin, 2018) [in Japanese].

[9] M. P. Kerr , S. Mensah , F. Besag , et al., “International Consensus Clinical Practice Statements for the Treatment of Neuropsychiatric Conditions Associated With Epilepsy,” Epilepsia 52, no. 11 (2011): 2133–2138. 21955156 10.1111/j.1528-1167.2011.03276.x

[10] N. Agrawal and M. Mula , “Treatment of Psychoses in Patients With Epilepsy: An Update,” Therapeutic Advances in Psychopharmacology 9 (2019): 2045125319862968. 31316747 10.1177/2045125319862968 PMC6620723

[11] Y. Calle‐López , L. D. Ladino , V. Benjumea‐Cuartas , D. M. Castrillón‐Velilla , J. F. Téllez‐Zenteno , and P. Wolf , “Forced Normalization: A Systematic Review,” Epilepsia 60, no. 8 (2019): 1610–1618. 31260102 10.1111/epi.16276

[12] J. A. Bragatti , “Forced Normalization Revisited: New Concepts About a Paradoxical Phenomenon,” Frontiers in Integrative Neuroscience 15 (2021): 736248. 34512281 10.3389/fnint.2021.736248 PMC8429494

[13] K. Kanemoto , T. Nishida , and N. Hasegawa , “Psychiatric Symptoms of Patients With Epilepsy: Characteristics of Psychiatric Adverse Events by Novel Antiepileptic Medications,” Brain and Nerve 75, no. 4 (2023): 375–389. 37037510 10.11477/mf.1416202343

[14] I. G. McKeith , B. F. Boeve , D. W. Dickson , et al., “Diagnosis and Management of Dementia With Lewy Bodies: Fourth Consensus Report of the DLB Consortium,” Neurology 89, no. 1 (2017): 88–100. 28592453 10.1212/WNL.0000000000004058 PMC5496518

[15] F. M. E. Pinckaers , M. E. Boon , and M. H. J. M. Majoie , “Risk Factors Predisposing to Psychotic Symptoms During Levetiracetam Therapy: A Retrospective Study,” Epilepsy & Behavior 100, no. Pt A (2019): 106344. 31525554 10.1016/j.yebeh.2019.05.039

[16] G. Zaccara and V. Franco , “Pharmacokinetic Interactions Between Antiseizure and Psychiatric Medications,” Current Neuropharmacology 21, no. 8 (2023): 1666–1690. 35611779 10.2174/1570159X20666220524121645 PMC10514545

[17] M. Agrawal , S. A. Borkar , and S. S. Kale , “Phenytoin Toxicity Manifesting as Acute Psychosis: An Uncommon Side Effect of a Common Drug,” Asian Journal of Neurosurgery 14, no. 2 (2019): 532–534. 31143275 10.4103/ajns.AJNS_86_18 PMC6516005

[18] J. A. J. Boermans and J. R. Nijdam , “Neurologic and Psychiatric Symptoms Following Chronic Phenytoin Intoxication,” Acta Neuropsychiatrica 12, no. 2 (2000): 46–48. 26976757 10.1017/S0924270800035699

[19] M. Bloechliger , S. Rüegg , S. S. Jick , C. R. Meier , and M. Bodmer , “Antipsychotic Drug Use and the Risk of Seizures: Follow‐Up Study With a Nested Case‐Control Analysis,” CNS Drugs 29, no. 7 (2015): 591–603. 26242478 10.1007/s40263-015-0262-y

[20] M. Habibi , F. Hart , and J. Bainbridge , “The Impact of Psychoactive Drugs on Seizures and Antiepileptic Drugs,” Current Neurology and Neuroscience Reports 16, no. 8 (2016): 71. 27315249 10.1007/s11910-016-0670-5

